# Identification of a de novo Mutation in TMEM106B in a Saudi Child Causes Hypomyelination Leukodystrophy

**DOI:** 10.1055/s-0043-1764370

**Published:** 2023-03-20

**Authors:** Lena Alotaibi, Amal Alqasmi

**Affiliations:** 1 Collage of Medicine, King Saud bin Abdul-Aziz University for Health Sciences, Riyadh, Saudi Arabia; 2 Department of Pediatric Neurology and Epilepsy, King Saud Medical City, Riyadh, Saudi Arabia

**Keywords:** TMEM106, Exome, hypomyelinating leukodystrophies, mutation

## Abstract

Hypomyelinating leukodystrophies are one of the white matter disorders caused by a lack of myelin deposition in the central nervous system (CNS). Here, we report the first case of hypomyelinating leukodystrophy in the Middle East and Saudi Arabia. This condition is caused by a mutation in the TMEM106B gene (HLD16; MIM 617964). Hypotonia, congenital nystagmus, delayed motor development, and delayed speech are the main clinical manifestations. The affected patient has mild pyramidal syndrome, a mild intellectual disability, ataxic gait, hyperreflexia, intention tremor, dysmetria, and other motor difficulties. Findings from neuroimaging reveal severe, ongoing, and diffuse hypomyelination identified via the whole exome sequencing, a harmful missense mutation in the TMEM106B gene that is heterozygous. The patient is the offspring of two unrelated persons. The protein's cytoplasmic domain contains a variation that is located in highly conserved residues. In an oligodendroglial cell line, the mutant protein significantly lowered the mRNA production of important myelin genes, decreased branching, and increased cell mortality. TMEM106B is abundantly expressed in neurons and oligodendrocytes in the CNS and is localized in the late endosome and lysosome compartments. TMEM106B levels can be controlled at the transcriptional level through chromatin modification, at the mRNA level through miRNAs, and at the protein level through lysosomal functions. Our findings reveal a novel role of zinc homeostasis in oligodendrocyte development and myelin production and show that variations in TMEM163 induce hypomyelination leukodystrophy.

## Introduction

A category of genetically diverse diseases known as hypomyelinating leukodystrophies is characterized by impaired myelination. About 15 years ago, magnetic resonance imaging (MRI) identified the characteristics of discrete disease entities. The diagnosis of genetic leukodystrophies has grown recently as a result of the advancement of molecular genetic testing techniques. 1 2

Since the discovery of Pelizaeus–Merzbacher disease (PMD [MIM 312080]) and the link with PLP1 mutations, more than 10 distinct hypomyelination leukodystrophies have been diagnosed and described. White matter integrity and function are determined by all of its elements, according to developed knowledge of the variety of white matter pathology that underlies leukodystrophies. 3 In the last 10 years, it has become clear that variations in gene coding for structural oligodendrocyte proteins, as well as variations in gene coding for transcription factors that regulate the expression of structural genes and other genes with cellular roles less directly related to oligodendrocyte function, may also be involved in hypomyelination leukodystrophies. 4

According to research on various white matter disorders that cause leukodystrophies, the integrity and function of white matter are determined by all of its elements. Over the past 10 years, it has become increasingly clear that genes other than those that code for structural oligodendrocyte proteins, as well as genes that code for transcription factors that govern the expression of those genes, may also play a role in hypomyelination leukodystrophies. 5

Hypomyelination leukodystrophies are caused by mutations in the gene encoding structural oligodendrocyte proteins like PLP1 or TUBB4A in addition to mutations in the SOX10 and NKX6-2 transcription factor genes 5 6 that govern the expression of structural genes. Additionally, mutations in gene coding for the tRNA synthetases RARS and DARS, POLR3A, POLR3B, and POLR1C, all of which are subunits of RNA polymerase 3, have been associated with changes in oligodendrocyte function. 7

Transmembrane protein 106B (TMEM106B; 7 p21) is a member of the TMEM106 family. Recently, hypomyelination leukodystrophy, neurodegenerative disorders, and brain aging have all been linked to TMEM106B, which codes for a lysosome membrane protein that is implicated in the trafficking of late endosomes and lysosomes in neurons.

Here we describe the case of a child with hypomyelination leukodystrophy in whom heterozygous de novo missense mutant variation in TMEM106B was discovered using exome sequencing.

## Clinical Report

The affected individual was of Arab descent. He, now 5 years old, is a child who presented at the age of 3 years in the clinic with horizontal nystagmus of both eyes and hypotonia with elevated muscle tone in his legs and increased tendon reflexes. He had a history of delayed development (sitting at 1.5 years and walking at 2.10 years), but at the age of 5, he could run and climb the stairs without holding on to the banisters. Fine motor skills were normal and he had age-appropriate receptive language skills, but his pronunciation and grammar in expressive language were poor. He could construct four- to six-word sentences. His IQ was not tested. Hearing was normal and there was no history of seizure. At the age of 5 years, a neurological examination revealed that he was not dysmorphic, but had dysarthria, gait ataxia, and dysmetria. Muscle bulk and tone were normal. Deep tendon reflexes were brisk with low plantar reflexes. He had rapid, bilateral nystagmus and no involvement of internal organs. His father, who was 42 years old, was in good health, as was his mother (34 years old). There was no history of abortion or death. He was the first child.

The metabolic and biochemical markers used in the diagnostic workup were normal, as were the lactic acid and quantitative amino acids. Neuroimaging was performed, including an MRI of the brain (see Fig. 1 ) performed at the age of 4 years, and showed clear characteristics of hypomyelination with a discrepancy between T1- and T2-weighted images. Increased white matter signal intensity is seen on T2 and fluid-attenuated inversion recovery (FLAIR) images in the supra- and infratentorial brain, involving the external and internal capsules as well as the corticospinal tract in the brainstem, indicating extensive demyelination.

**Figure:**
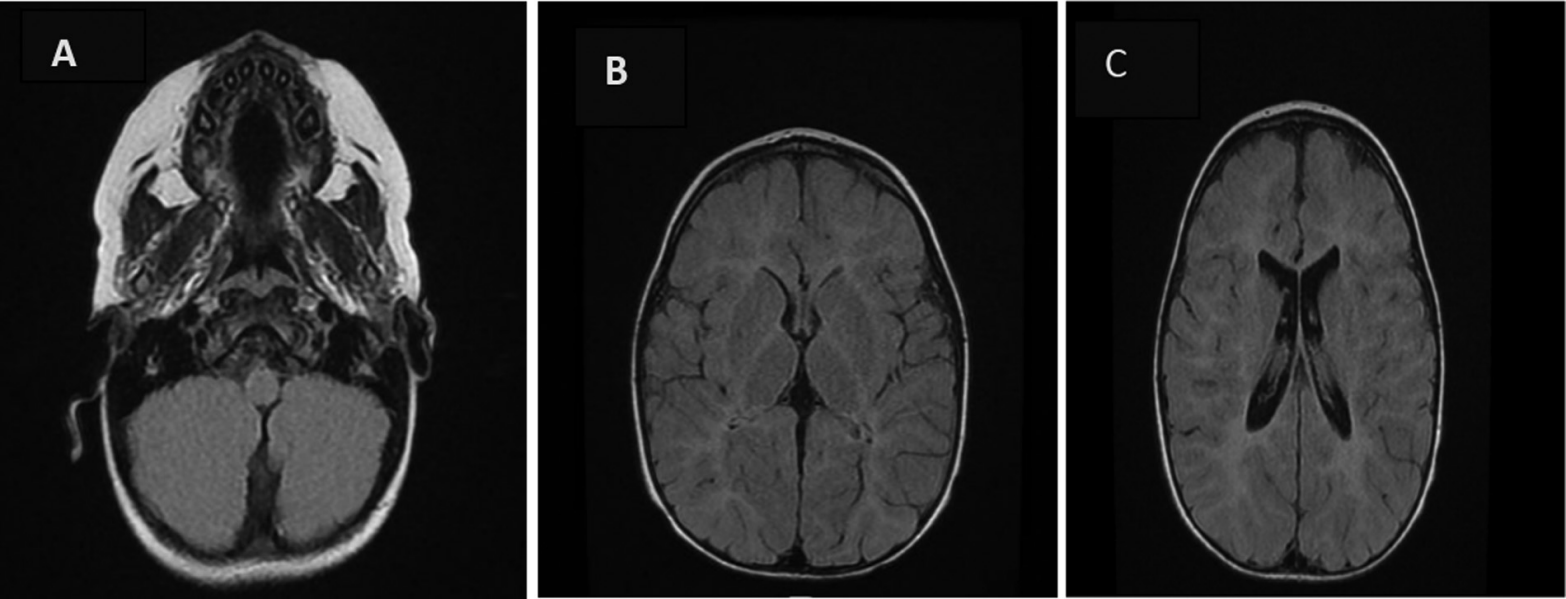
Fig. 1 (A–C) Brain magnetic resonance imaging (MRI) at the age of 4 years showed hypomyelination with a discrepancy between T1- and T2-weighted images and increased white matter signal intensity is seen on T2 and fluid-attenuated inversion recovery (FLAIR) images in the supra- and infratentorial brain involving the external and internal capsules as well as the corticospinal tract in the brainstem, indicating extensive demyelination.

## Discussion

We describe the case of a child with hypomyelinating leukodystrophy who had de novo missense variations in TMEM106B that were discovered by exome sequencing. This child had strong genotypic and phenotypic features of hypomyelinating leukodystrophy. He exhibited a clinically early-onset hypotonia with ataxia of gait and dystonia, along with a pronounced horizontal nystagmus. Mild intellectual disabilities existed. According to the description of the hypomyelinating leukodystrophy, there was a difference between the T1- and T2-weighted images on cranial MRI.

Hypomyelinating leukodystrophies have been described in previous cases. They were alive at 19, 5, 38, and 26 years despite having early-onset nystagmus and hypotonia. Early childhood characteristics included delayed motor development, hypertonia and hyper-reflexes, and poor nutrition, dystonia, wide-based or ataxic gait, dysarthria, dysmetria, saccadic pursuit, and tremor. The most severely affected patient began walking at the age of 13 years and was verbally nonfunctional at the age of 19 years, whereas the other patients began walking between the ages of 3 and 5 years. Only two patients who were treated for the cognitive performance variable had seizures. In the majority of patients, hypomyelination was visible on MRI, and one elderly patient had a thin corpus callosum. A comparison of the geno- and phenotypic features of our patient and the other patients who have been reported is presented in Table 1 .

**Table 1:** Geno- and phenotypic features of our patient and the other patients who have been reported

Patient	Our case	1	2	3	4	5
TMEM106B-related variants	c.754G > A p.(Asp252Asn)	c.754G4A p.(Asp252Asn)	c.754G4A p.(Asp252Asn)	c.754G4A p.(Asp252Asn)	c.754G4A p.(Asp252Asn)	c.745G4A (p.D252N)
Variant type	Missense	Missense	Missense	Missense	Missense	Missense
Ethnicity	Saudi	European	European	European	European	Chinese
Inheritance	De novo	De novo	De novo	AD	De novo	De novo
Sex	Male	Male	Male	Female	Male	Female
Age (y)	5	19	5	38	26	3
Gross motor delay	+	+	+	+	+	+
Speech	Speech delay	Nonverbal	Speech delay	Speech delay	Speech delay	Speech delay
Intellectual disability	Mild	Severe	Moderate	Mild	Mild	Moderate
Seizures	–	–	–	+	+	–
Gait	Ataxia	–	Ataxia	Ataxia	Mild ataxia	–
Eyes	Nystagmus (horizontal)	Nystagmus	Nystagmus (horizontal, vertical)	Nystagmus (pendular, horizontal)	Nystagmus (rotatory)	Nystagmus (vertical, rotational)
Muscle tone	Decreased	Normal	Increased	Increased	Normal	Normal
Brain MRI	Diffuse hypomyelination	Hypomyelination	Hypomyelination	Hypomyelination Thin corpus callosum	Hypomyelination	Diffuse hypomyelination

Abbreviations: MRI, magnetic resonance imaging.

TMEM106B is a transmembrane protein containing 274 amino acids, glycosylated. The N-terminus is located in the cytosol. The C-terminus is located in the lumen is restricted to the membrane of late endolysosomal compartments. 8 It has a role in the transport of late endosomes and lysosomes in neurons and regulates lysosomal size, motility, and trafficking. 9 10

On chromosome 7p21, TMEM106B, a number of single-nucleotide polymorphisms (SNPs) were discovered in significant linkage disequilibrium, leading to the identification of two widely distributed TMEM106B haplotypes in the human population. 11 Of these two haplotypes, it is the one that has been consistently linked to a higher risk of neurodegenerative disorders and poor mental health. However, there has been much debate regarding the disease-modifying variation to blame as well as the functional impact of the risk haplotype.

Genomic DNA is enzymatically fragmented, and mitochondrial genome-specific DNA-captured probes are used for enrichment. The libraries are subsequently sequenced on an Illumina platform to achieve at least 200x depth of coverage for 97% of the target region. Data analysis, including read alignment to the Revised Cambridge Reference Sequence (rCRS) of the Human Mitochondrial DNA (NC_012920), variant calling, and annotation are performed using validated in-house software. The pipeline confidently detects the heteroplasmy levels down to 5%. Copy number variations are detected based on read depth. All identified variants are evaluated with respect to their pathogenicity and causality.

Double-stranded DNA capture baits against approximately 36.5 Mb of the human coding exome (targeting >98% of the coding RefSeq from the human genome build GRCh37/hg19) are used to enrich target regions from fragmented genomic DNA with the Twist Human Core Exome Plus kit. The generated library is sequenced on an Illumina platform to obtain at least 20x coverage depth for >98% of the targeted bases. An in-house bioinformatics pipeline, including read alignment to GRCh37/hg19 genome assembly, variant calling, annotation, and comprehensive variant filtering, is applied. All variants with a minor allele frequency (MAF) of less than 1% in the gnomAD database and disease-causing variants reported in HGMD, ClinVar, or CentoMD are considered. The investigation for relevant variants is focused on coding exons and flanking ± 20 intronic nucleotides of genes with clear gene-phenotype evidence (based on OMIM information).

It revealed the TMEM106B variant c.754G > A p. (Asp252Asn) causes an amino acid change from Asp to Asn at position 252. According to HGMD Professional 2020.1, this variant has previously been described as disease-causing for hypomyelinating leukodystrophy by Simons et al (PMID: 29186371), who reported this variant in four unrelated patients with brain hypomyelination as de novo in three of the cases, and the mildly affected father of the fourth affected individual was confirmed as a mosaic for this variant. Yan et al (PMID: 29444210) reported this variant as de novo through a triage analysis in one patient. It is classified as pathogenic (class 1) according to the recommendations of CENTOGENE and ACMG.

The TMEM106B variant c.754G > A p.(Asp252Asn), which was identified by trio-based whole-exome or whole-genome sequencing, occurred de novo in most cases reported, but the mildly affected father of one adult female was mosaic for the mutation. The most severely affected patient reported by [Simon] has carried a potentially damaging de novo missense variant in the USP7 gene, which may have contributed to his phenotype.

In conclusion, we describe the first Saudi patient with an autosomal dominant hypomyelinating leukodystrophy and a heterozygous de novo missense variation in TMEM106B. We show evidence that an Asp to Asn substitution in TMEM106B results in CNS hypomyelination with a mild clinical course. This evidence comes from the consistent clinical phenotype and MRI pattern of hypomyelinating illnesses.
